# Validation of an NGS mutation detection panel for melanoma

**DOI:** 10.1186/s12885-017-3149-0

**Published:** 2017-02-22

**Authors:** Anne Reiman, Hugh Kikuchi, Daniela Scocchia, Peter Smith, Yee Wah Tsang, David Snead, Ian A Cree

**Affiliations:** 1 Department of Pathology – Coventry and Warwickshire Pathology Services (CWPS), University Hospitals Coventry and Warwickshire, Coventry, CV2 2DX UK; 2 Centre for Research in Applied Biological and Exercise Sciences, Coventry University, Coventry, CV1 5FB UK; 3 Institute of Ophthalmology, University College London, Bath Street, London, EC1V 9EL UK; 4 Centre for Technology Enabled Health Research (CTEHR), Faculty of Health & Life Sciences, Coventry University, Coventry, CV1 5FB UK

**Keywords:** Melanoma, NGS, PCR, Mutation, BRAF, NRAS

## Abstract

**Background:**

Knowledge of the genotype of melanoma is important to guide patient management. Identification of mutations in BRAF and c-KIT lead directly to targeted treatment, but it is also helpful to know if there are driver oncogene mutations in NRAS, GNAQ or GNA11 as these patients may benefit from alternative strategies such as immunotherapy.

**Methods:**

While polymerase chain reaction (PCR) methods are often used to detect BRAF mutations, next generation sequencing (NGS) is able to determine all of the necessary information on several genes at once, with potential advantages in turnaround time. We describe here an Ampliseq hotspot panel for melanoma for use with the IonTorrent Personal Genome Machine (PGM) which covers the mutations currently of most clinical interest.

**Results:**

We have validated this in 151 cases of skin and uveal melanoma from our files, and correlated the data with PCR based assessment of BRAF status. There was excellent agreement, with few discrepancies, though NGS does have greater coverage and picks up some mutations that would be missed by PCR. However, these are often rare and of unknown significance for treatment.

**Conclusions:**

PCR methods are rapid, less time-consuming and less expensive than NGS, and could be used as triage for patients requiring more extensive diagnostic workup. The NGS panel described here is suitable for clinical use with formalin-fixed paraffin-embedded (FFPE) samples.

**Electronic supplementary material:**

The online version of this article (doi:10.1186/s12885-017-3149-0) contains supplementary material, which is available to authorized users.

## Background

**Fig. 1:**
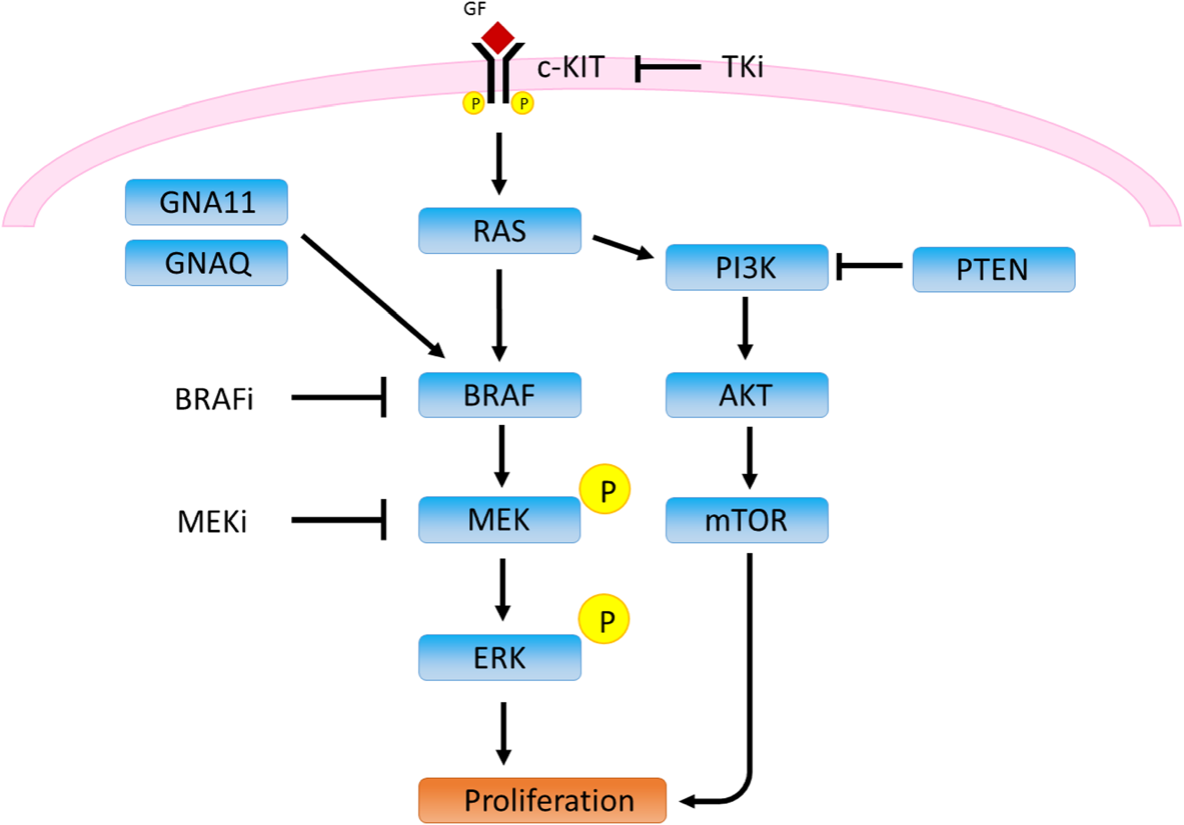
Growth pathway in melanoma, showing the genes included in the IonTorrent panel which can be targeted by tyrosine kinase inhibitors

Driver genetic abnormalities within growth pathways can be identified in most tumour types and define response to targeted anti-cancer drugs. In melanoma, validated drug targets with companion diagnostic utility now include BRAF and c-KIT, as these are driver oncogenes in which activating mutations occur (Fig. 1) and can be targeted by tyrosine kinase inhibitors (TKIs). Demonstration of other mutations in other driver oncogenes, particularly NRAS, GNAQ and GNA11, can be useful in determining treatment strategy, such as an early decision to use immunotherapy with CTLA4 or PDL1 inhibitors. The number of drugs and the complexity of their associated companion diagnostic pathways is increasing rapidly. Additional information from gene and protein expression can also assist comprehensive analysis of melanoma biology [1, 2].

**Fig. 2:**
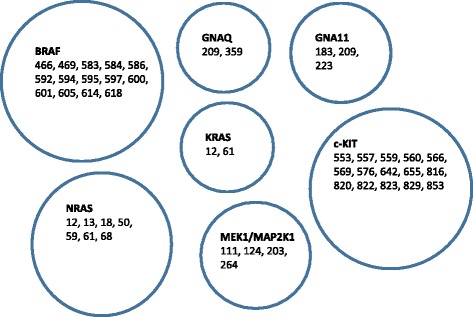
Diagram showing the codons for each gene within the Ion Torrent melanoma panel: the majority of mutations tested were within BRAF, NRAS and c-KIT

Pathology laboratories need to provide a rapid, reliable and comprehensive diagnostic molecular pathology service to oncologists [3]. This is challenging, particularly when diagnostics budgets are under pressure. However, advances in the automation of DNA and RNA retrieval from FFPE samples, as well as improvements in immunohistochemistry mean that tumours can be characterised rapidly for mutational status [4].

There are a large number of commercial methods based on polymerase chain reaction (PCR) available for BRAF determination in melanoma. Those most commonly used include cobas® (Roche Diagnostics, Burgess Hill, UK) and Therascreen® (Qiagen, Manchester, UK). Many laboratories still use older sequencing technologies including Sanger and Pyrosequencing, and the use of NGS methods is becoming more common. Different methods tolerate different degrees of DNA quality, require different levels of operator skill, and need different levels of expertise for their interpretation [5]. Despite this, comparative studies are relatively uncommon. A recent study compared Sanger sequencing with cobas® for BRAF V600E mutation [6]. The detection rate did not differ significantly, but six cases were missed by cobas which does not cover all of the mutations potentially present. Conversely, while cobas PCR produced results in every case tested, Sanger sequencing failed in ten cases [6]. Sanger sequencing usually has lower sensitivity than targeted PCR, but the assays also have different requirements for input DNA. Sensitivity is not the only issue: the amount of input DNA required varies between technologies and can have a major effect on the feasibility of testing small samples. The BRAF cobas test requires 125 ng input DNA, while the Sanger sequencing method used in the study cited above [6] needed just 50 ng DNA [7]. TaqMan castPCR technology (ThermoFisher, Paisley, UK) has been shown comparable to pyrosequencing [8], and requires 50 ng of DNA per reaction in 96 well plates (200 ng in total), but only 100 ng DNA per 48 wells in Taqman array format [9], while Therascreen requires 80 ng DNA per reaction (320 ng in total) [10]. The simplicity of the PCR method chosen is also important to busy routine pathology laboratories, where the availability of expert staff is a major factor [5].

We recently participated in the validation of a 22 gene Ampliseq panel for lung and colorectal cancer on the IonTorrent Personal Genome Machine (PGM) (ThermoFisher) [11], and have now designed a melanoma panel covering all actionable melanoma mutations, though it would also be feasible to use validated comprehensive gene panel if this is preferred. Despite recent improvements, NGS is challenging to use in a routine molecular pathology environment and many laboratories still prefer a PCR solution. Taqman Array™ technology (ThermoFisher) can be used with castPCR mutation detection assays to provide an alternative and less expensive panel testing facility to NGS methods [9]. Our current panel incorporates KRAS, NRAS, EGFR, and BRAF for combined screening of common mutations in colorectal cancer, lung cancer and melanoma, known as the REB array [9].

While multiple gene PCR is helpful, there is also a need for validated NGS solutions suitable for clinical use to assist the molecular characterisation of melanoma, particularly when these do not identify driver mutations. We therefore compared our Ampliseq panel for use on the IonTorrent PGM NGS platform with routine PCR methods and the recently validated REB array.

## Methods

The study was designed as a direct comparison between multi-gene PCR (TaqMan™ array) and (IonTorrent) NGS based against routine Therascreen® or cobas® testing for BRAF alone.

### Study population

A total of 151 samples were sourced from 2014 and 2015 from 132 melanoma patients attending University Hospital Coventry and Warwickshire (UHCW) for diagnosis and treatment, including 18 uveal melanomas blocks obtained from the Institute of Ophthalmology London for comparison. All advanced melanomas are routinely tested for BRAF, either in house, or at a referral centre using Therascreen® (Qiagen, Manchester UK) or cobas® (Roche Diagnostics, Burgess Hill, UK). Clinical data supplied with the specimens were collected from the Pathology Laboratory Information Management System (ULTRA, Cirdan, Lisburn, Northern Ireland, UK). To ensure that all melanoma types can be handled, we have included 2 mucosal and 4 acral lentiginous melanomas, as well as 18 uveal melanomas.

### DNA extraction

Areas of high cellularity melanoma (>50% neoplastic cells) were marked on a slide by a histopathologist. These were matched with the corresponding block. Samples from the areas marked were then punched out using a 1 mm diameter skin punch with plunger (Integra Miltex, Plainsboro, NJ, USA). DNA extraction was performed using the Promega Maxwell™ instrument, according to the manufacturer’s instructions. DNA content and quality was checked by Qubit 2.0 fluorimeter (ThermoFisher) according to the manufacturer’s instructions using their HS dsDNA kit before mutation detection. We used a volume of 1 μl (less than 50 ng DNA) to conserve as much of the sample as possible, and worked with a range of concentrations, depending on the extraction concentration achieved.

### REB Taqman array

CastPCR™ provides a platform for sensitive mutation detection and in combination with TaqMan Array provides a robust solution with minimal pipetting steps. Extracted DNA (100 ng) is diluted with Taq polymerase and added to a port of a manufactured microfluidic array [9]. TaqMan arrays can be run on AB7900HT, ViiA7 and Quantstudio PCR machines (ThermoFisher), although for this study, we used the ViiA7 instrument. The REB array method requires little operator time or experience: it covers all BRAF mutations of relevance and >95% of reported NRAS mutations.

### IonTorrent NGS

The panel design was based on the Catalogue of Somatic Mutations in Cancer (COSMIC) database and literature searches, and included seven genes important in melanoma tumorigenesis (Fig. 2, Table 1 and Additional file 1: Table S1). A total 95 hotspot mutations in these genes were submitted to the Ampliseq designer tool (https://ampliseq.com/browse.action. The resultant primer pool consisted of 21 primer pairs with 98% of predicted coverage of the target region (hotspots COSM1139 and COSM1142 were not included in the final design). The final target region was 2.47kb with the amplicon range 125-175 bp in length. The codons listed for each gene included in the Ampliseq panel are shown in Table 1.

**Table 1:** Summary of custom Ampliseq gene panel for melanoma, including common driver mutations in BRAF, KRAS, NRAS, MEK, GNAQ, and GNA11

Gene	Reference	Codons included	Number of Mutations
BRAF	NM_004333.4	466, 469, 583, 584, 586, 592, 594, 595, 597, 600, 601, 605, 614, 618	29
GNAQ	NM_002072.3	209, 359	6
GNA11	NM_002067.2	183, 209, 223	3
NRAS	NM_002524.4	12, 13, 18, 50, 59, 61, 68	7
c-KIT	NM_000222.2	553, 557, 559, 560, 566, 569, 576, 642, 655, 816, 820, 822, 823, 829, 853	18
KRAS	NM_033360	12, 61	5
MEK1/MAP2K1	NM_002755.2	111, 124, 203, 264	5

Sequencing was performed as previously described [11], with the following changes to accommodate IonChef loading of the IonTorrent 314 chips. 10 ng of gDNA from each of the samples chosen for NGS analysis was combined with the Ampliseq™ reagents and primer pool for the melanoma gene panel and amplified initially for 23 cycles, which was later optimised to 28 cycles. After initial amplification, the amplified products were partially digested before IonExpress Adapters and Barcode sequences were ligated to the library fragments. Following barcoding the libraries were cleaned up using a magnetic bead method (IonTorrent, ThermoFisher) and then quantified by the Ampliseq Q-PCR method (ThermoFisher). Once the libraries were successfully quantified they were combined and diluted to 50 pM. For IonTorrent 314 chips, 7 libraries on average were combined per chip. These library pools were then loaded into the IonChef instrument for further library preparation and chip loading. The loaded 314 chips were then run on the IonTorrent PGM instrument according to manufacturer’s instructions. The Variant Caller plugin (included in the provided Ion Torrent Suite software version 4.2) was used to analyse the aligned sequence data for the identification of hotspot mutations and novel variants. The variant caller files were subjected for further analysis with the Ion Reporter online tool. Coverage was regarded as acceptable if mean was at least 300×, and the Variant caller default setting interrogating somatic variants with high stringency was used for hotspot calling, to exclude those < 2% allele frequency. Only known mutations with COSMIC references were included. For the purposes of this analysis, SNPs and variants of unknown significance were ignored.

### Data and economic analysis

A sample size of 150 biopsies is expected to be sufficient to meet the need for comparison of the two methods, based on an expected Kappa of 0.9, with a 2-tailed Null value of 0.6 and 80% power. Data from both PCR (all methods) and NGS were compared using kappa statistics, and discrepant results tested independently by another laboratory. Costing data were obtained from timesheets and invoice information held within the pathology laboratory for use with the CMD Impact tool available on-line from the Royal College of Pathologists (www.rcpath.org/cmd-impact.html).

### Ethics

The development of service improvements does not require specific research ethics committee approval as stated in the EU Clinical Trials Directive (2001/20/EC). However, in this instance, we obtained most samples from the Arden Tissue Bank, (ATB15-001) which has NRES ethics approval (12/SC/0526) to conduct studies on anonymised samples that are not required for diagnosis. In practice, written consent for use of tissue was obtained from all patients as part of the surgical consent process.

## Results

A total of 151 blocks from 148 resections were submitted for NGS testing, representing 132 patients with repeat samples available from metastases and primary tumours in 11 patients. There were 76 males and 37 females with cutaneous melanoma. The average age of the patients was 66 years (range 22 - 95 years, median 70 years). Duplicate blocks were tested from the same sample for three samples repeated as controls, producing the same results in each case.

### Patients

A total of 105 patients had cutaneous melanoma (including 4 acral lentiginous melanoma and 2 malignant blue naevus), 2 had mucosal melanoma, 19 uveal melanomas (18 primary, 1 metastatic), and 6 unknown primary site.

### Mutations

The mutations identified in each group are summarized in Table 2. Driver mutations were identified in 70% of the patients studied, and in 74% of the cutaneous melanomas (all types). BRAF was the commonest mutation found, present in 44 of the 105 cutaneous patients (42%). NRAS was present in 28 of the 105 cutaneous melanomas (27%), and small numbers of KIT and MEK1 mutations were identified in cutaneous melanomas (3 and 4 cases respectively). Both mucosal melanomas tested were wild-type, but 4 of the 6 melanomas of unknown primary origin had mutations, including one GNAQ mutation suggesting that this might have been of uveal origin. Mutations were found in 10 of the 19 uveal melanomas, with equal numbers of GNA11 and GNAQ mutations, but there were also two NRAS mutations.

**Table 2:** Summary of mutations identified by melanoma type

Melanoma Type	BRAF	NRAS	KRAS	KIT	MEK	GNA11	GNAQ	WT	Total
Cutaneous SSM	15	11	0	1	1	0	0	6	34
Cutaneous Nodular	15	11	0	0	1	0	0	11	38
Cutaneous NOS	12	3	0	1	1	0	0	5	22
LMM	1	2	0	0	0	0	0	2	5
Acral Lentiginous	0	1	0	0	1	0	0	2	4
Malignant blue naevus	1	0	0	0	0	0	0	1	2
Mucosal	0	0	0	0	0	0	0	2	2
Unknown Primary	0	3	0	1	0	0	1	1	6
Uveal	0	2	0	0	0	4	4	9	19
Total	44	33	0	3	4	4	5	39	132

### BRAF correlation

No molecular pathology was done routinely by the pathologist in 33 cases, mainly due to the presence of intercurrent disease, and the 19 historical uveal melanoma cases. In the remaining 80 cases, molecular investigation for BRAF mutations was done by cobas PCR. For BRAF, patient-based correlation with cobas detection in 80/132 cases was 92.5%, with six discrepancies, as shown in Table 3. For the 33 cases without cobas, the REB array was run with residual DNA from the NGS sample. The assay failed in 2 cases, but resulted in a further 15 cases with complete correlation BRAF mutation between PCR and NGS, including 2 cases with V600K mutations: the remainder were confirmed as wild-type (100% correlation).

**Table 3:** Discrepancies between cobas PCR for BRAF and NGS. As cobas does not distinguish mutations at BRAF codon 600, these are designated V600X in the table, WT is wild-type

Case (Sample)	cobas	NGS	Comment	True discrepancy
30	WT	BRAF	Mutation not present on cobas	Yes
33	WT	BRAF	Mutation not present on cobas	Yes
35	WT	BRAF	Mutation not present on cobas	Yes
48	V600X	MEK1	Resistance gene due to vemurafenib treatment	No
85	V600X	NRAS	Resistance gene due to vemurafenib treatment	No
100	V600X	KIT	Resistance gene due to vemurafenib treatment	No

### Other mutations

NRAS is present on the REB array (though not on cobas) and was completely concordant in 5 samples found to have the Q61R mutation founds by NGS. Three KIT mutations were identified (Table 2), and correlated with known KIT mutations by pyrosequencing performed at University Hospital Birmingham in 2 cases, which was from a skin primary and melanoma from an unknown primary site. MEK mutation was seen in 1 case, which was under treatment with a BRAF inhibitor (vemurafenib), and a KIT mutation was identified in another case on treatment (Table 3).

The costs of NGS are compared with the PCR Taqman Array previously published in Table 4, showing that PCR is less expensive at £135 per case than NGS at £257 per case. There are differences in the cost of reagents, but the major difference in costs is due to the staff costs for NGS (£ per case) in comparison with PCR (£ per case).

**Table 4:** Costs of the Taqman PCR array per sample for mutation analysis [9], versus IonTorrent PGM analysis using the Ampliseq method for our laboratory. The major differences are due to the high cost of consumables for NGS

Cost (£/sample)	PCR Array (per sample)	NGS panel (per sample)
Cost of consumables per test	40	134
External quality assurance	6	5
Equipment rental and maintenance	35	45
Staff costs	54	73
Total per sample	135	257

## Discussion

Our results validate the use of a custom design targeted Ampliseq panel for melanoma mutation detection. Our panel includes the genes commonly mutated in ocular (uveal) and mucosal melanoma as well as the more common cutaneous tumours. The panel described here has been used in one patient with metastatic melanoma who had a history of both skin and uveal primary lesions. The clinical question being which one had given rise to his metastatic disease. The sample contained a GNA11 mutation, indicative of metastasis from the uveal tumour.

We provide further evidence for the utility of the REB array in melanoma, and this could be used to triage samples for the more expensive NGS method, which has a longer turnaround time (90 min compared with 2 days, from DNA to result). However, it should be noted that rapid turnaround is of limited clinical relevance to melanoma patients, unless they present with widespread metastases.

The ability of NGS to look for MEK mutations associated with resistance to vemurafenib may be helpful in guiding patient treatment, and there is a case to be made for re-testing of samples taken on progression. In this series, only two patients had documented resistance, one of whom developed a MEK mutation, while the other had a KIT mutation, both of which are potentially treatable.

Ampliseq™ provides targeted NGS for hotspots. The NRAS and BRAF primers used have been previously validated with the 22 gene Onconetwork IonTorrent PGM panel which includes both [11]. For known mutations, results from variant caller can be obtained directly or uploaded to IonReporter for more detailed analysis. We chose the former approach, due to security of data concerns which prevent us from routinely uploading data to IonReporter over non-hospital systems. However, the default IonTorrent analysis settings worked well in this study and variant caller does provide COSMIC references for known variants, allowing rapid checking of mutations for individual samples.

Comparison of different methods is important, but can be complicated. Each method designed to identify actionable mutations or hotspots, rather than to sequence entire genes, has slightly differing coverage, and differing sensitivity for each mutation included. Discrepancies between methods are therefore inevitable and expected. Investigation usually reveals that such discrepancies often involve rare variants or variants of unknown significance. We have identified a number of comparative studies in the literature, but perhaps less than one might expect given the number of methods commercially available, let alone those used as in-house laboratory-developed (‘homebrew’) tests. The majority of these compare in-house tests with Sanger sequencing and commercially available PCR assays, with some variation in sensitivity, but few discrepancies overall [4, 6, 12–16]. There are the expected issues with coverage: for instance, some PCR methods do not detect the relatively common V600K mutation [16]. If there is a clinical need to identify other mutations than those tested, the use of cascade testing with other methods should be considered. We now use the REB array [9] as a triage for NGS: those patients who show a BRAF or NRAS mutation can be excluded from further testing, while those in whom it is clinically relevant to identify other driver mutations (e.g. c-KIT) can be pursued further.

The costs of the two methods differ substantially (Table 4). As expected, the REB array proved much less expensive than NGS, but also has less coverage. In reality, the REB array can be used to exclude tumours with common BRAF or NRAS mutations from further testing, allowing resources to be concentrated on sequencing cases where the driver mutation is not known, and knowledge of the KIT status of the tumour may be helpful. This is not a large panel, and we rarely require NGS on more than 8 patients at a time, and we therefore selected the 314 chip as optimal. The number of cases could be scaled up for the 316 and 318 chips, but this is unlikely to alter the cost per patient. The degree of automation now available using the IonChef™ for chip loading releases operator time, but library preparation is not yet automated and NGS does need considerable molecular pathology expertise. This is the major source of the increased cost of NGS in comparison with PCR methods, and should decline as automation is introduced.

## Conclusion

The methods compared here all have the ability to find mutations within melanoma, particularly those in BRAF. We have found it helpful to know of NRAS mutations to manage resources, and increasingly to direct patients towards immunotherapy at an earlier stage of their management. Those without an identified driver mutation may well benefit from the NGS approach used here, or a more comprehensive panel, depending upon workflow within the laboratory.

## Additional file

Additional file 1: Table S1. Complete mutation hotspot list included in Custom Ampliseq gene panel for melanoma, including common driver mutations in BRAF, NRAS, KRAS, MEK, GNAQ, and GNA11. (DOCX 125 kb)

## Abbreviations

BRAF: B-Raf proto-oncogene, serine/threonine kinase
COSMIC: Cataloque of somatic mutations in cancer database
CTLA4: cytotoxic T-lymphocyte-associated protein 4 (CD152)
DNA: Dexoxyribose nucleic acid
dsDNA: double stranded DNA
EGFR: Epidermal growth factor receptor
FFPE: Formalin-fixed and paraffin-embedded
GNA11: Guanine nucleotide-binding protein subunit alpha-11
GNAQ: Guanine nucleotide-binding protein G(q) subunit alpha
KIT: Mast/stem cell growth factor receptor (SCFR, CD117)
MEK: Dual specificity mitogen-activated protein kinase kinase 1
NGS: Next generation sequencing
PCR: Polymerase chain reaction
PDL1: Programmed death ligand 1 (B7-H1, CD274)
REB: RAS, EGFR and BRAF (array)

## References

[1] Jewell R, Conway C, Mitra A, Randerson-Moor J, Lobo S, Nsengimana J (2010). Patterns of expression of DNA repair genes and relapse from melanoma. Clin Cancer Res.

[2] Parker KA, Glaysher S, Polak M, Gabriel FG, Johnson P, Knight LA (2010). The molecular basis of the chemosensitivity of metastatic cutaneous melanoma to chemotherapy. J Clin Pathol.

[3] van Krieken JH, Normanno N, Blackhall F, Boone E, Botti G, Carneiro F (2013). Guideline on the requirements of external quality assessment programs in molecular pathology. Virchows Arch.

[4] Ehsani L, Cohen C, Fisher KE, Siddiqui MT (2014). BRAF mutations in metastatic malignant melanoma: comparison of molecular analysis and immunohistochemical expression. Appl Immunohistochem Mol Morphol.

[5] Cree IA, Deans Z, Ligtenberg MJ, Normanno N, Edsjo A, Rouleau E, et al. Guidance for laboratories performing molecular pathology for cancer patients. J Clin Pathol. 2014;67:923–931. 10.1136/jclinpath-2014-202404 PMC4215286 25012948

[6] Jurkowska M, Gos A, Ptaszynski K, Michej W, Tysarowski A, Zub R (2015). Comparison between two widely used laboratory methods in BRAF V600 mutation detection in a large cohort of clinical samples of cutaneous melanoma metastases to the lymph nodes. Int J Clin Exp Pathol.

[7] Rutkowski P, Gos A, Jurkowska M, Switaj T, Dziewirski W, Zdzienicki M (2014). Molecular alterations in clinical stage III cutaneous melanoma: correlation with clinicopathological features and patient outcome. Oncol Lett.

[8] Pisareva E, Gutkina N, Kovalenko S, Kuehnapfel S, Hartmann A, Heinzerling L (2014). Sensitive allele-specific real-time PCR test for mutations in BRAF codon V600 in skin melanoma. Melanoma Res.

[9] Kikuchi H. RA, Nyoni, J, Lloyd K., Savage R, Wotherspoon T, Berry L, Snead D, Cree IA. Development and validation of a Taqman array for cancer mutation analysis. Pathogenesis. 2016: In press.

[10] Garcia-Dios DA, Lambrechts D, Coenegrachts L, Vandenput I, Capoen A, Webb PM (2013). High-throughput interrogation of PIK3CA, PTEN, KRAS, FBXW7 and TP53 mutations in primary endometrial carcinoma. Gynecol Oncol.

[11] Tops B, Normanno N, Kurth H, Amato E, Mafficini A, Rieber N (2015). Development of a semi-conductor sequencing-based panel for genotyping of colon and lung cancer by the Onconetwork consortium. BMC Cancer.

[12] Ihle MA, Fassunke J, Konig K, Grunewald I, Schlaak M, Kreuzberg N (2014). Comparison of high resolution melting analysis, pyrosequencing, next generation sequencing and immunohistochemistry to conventional Sanger sequencing for the detection of p.V600E and non-p.V600E BRAF mutations. BMC Cancer.

[13] Machnicki MM, Glodkowska-Mrowka E, Lewandowski T, Ploski R, Wlodarski P, Stoklosa T (2013). ARMS-PCR for detection of BRAF V600E hotspot mutation in comparison with Real-Time PCR-based techniques. Acta Biochim Pol.

[14] Huang T, Zhuge J, Zhang WW (2013). Sensitive detection of BRAF V600E mutation by Amplification Refractory Mutation System (ARMS)-PCR. Biomark Res.

[15] Colomba E, Helias-Rodzewicz Z, Von Deimling A, Marin C, Terrones N, Pechaud D (2013). Detection of BRAF p.V600E mutations in melanomas: comparison of four methods argues for sequential use of immunohistochemistry and pyrosequencing. J Mol Diagn.

[16] Ahn S, Lee J, Sung JY, Kang SY, Ha SY, Jang KT (2013). Comparison of three BRAF mutation tests in formalin-fixed paraffin embedded clinical samples. Korean J Pathol.

