# Supplementary material for: Validation of an NGS mutation detection panel for melanoma
Source: BMC Cancer. 2017 Feb 22;17:150. doi: 10.1186/s12885-017-3149-0 (PMC5322598; doi:10.1186/s12885-017-3149-0)
Supplement: Additional file 1: Table S1. — Complete mutation hotspot list included in Custom Ampliseq gene panel for melanoma, including common driver mutations in BRAF, NRAS, KRAS, MEK, GNAQ, and GNA11. (DOCX 125 kb) [file 12885_2017_3149_MOESM1_ESM.docx]

Additional file 1: Table S1: Complete mutation hotspot list included in Custom Ampliseq gene panel for melanoma, including common driver mutations in BRAF, NRAS, KRAS, MEK, GNAQ, and GNA11.

| ***Gene*** | ***Reference*** | ***Mutation*** | ***Protein*** | ***COSMIC reference*** |
| --- | --- | --- | --- | --- |
|  |  |  |  |  |
| **BRAF** | **NM_004333.4** | c.1799T>A | p.V600E | COSM476 |
|  |  | c.1798_1799GT>AA | p.V600K | COSM473 |
|  |  | c.1798_1799GT>AG | p.V600R | COSM474 |
|  |  | c.1799_1800TG>AT | p.V600D | COSM477 |
|  |  | c.1798G>A | p.V600M | COSM1130 |
|  |  | c.1801A>G | p.K601E | COSM478 |
|  |  | c.1799T>C | p.V600A | COSM18443 |
|  |  | c.1799T>G | p.V600G | COSM6137 |
|  |  | c.1798G>C | p.V600L | COSM219798 |
|  |  | c.1799_1800TG>AA | p.V600E | COSM475 |
|  |  | c.1799_1801delTGA | p.V600_K601>E | COSM1133 |
|  |  | c.1789C>G | p.L597V | COSM470 |
|  |  | c.1789_1790CT>TC | p.L597S | COSM1126 |
|  |  | c.1790T>A | p.L597Q | COSM1125 |
|  |  | c.1781A>G | p.D594G | COSM467 |
|  |  | c.1780G>A | p.D594N | COSM27639 |
|  |  | c.1397G>A | p.G466E | COSM453 |
|  |  | c.1405G>A | p.G469R | COSM457 |
|  |  | c.1758A>G | p.E586E | COSM1118 |
|  |  | c.1749T>C | p.F583F | COSM1116 |
|  |  | c.1776A>G | p.I592M | COSM1119 |
|  |  | c.1406G>C | p.G469A | COSM460 |
|  |  | c.1750C>T | p.L584F | COSM28010 |
|  |  | c.1752T>C | p.L584L | COSM1117 |
|  |  | c.1784T>C | p.F595S | COSM1123 |
|  |  | c.1791A>G | p.L597L | COSM1124 |
|  |  | c.1813A>G | p.S605G | COSM21542 |
|  |  | c.1840T>C | p.S614P | COSM1139 |
|  |  | c.1853T>C | p.L618S | COSM1142 |
|  |  |  |  |  |
| **GNAQ** | **NM_002072.3** | c.626A>T | p.Q209L | COSM28757 |
|  |  | c.626A>C | p.Q209P | COSM28758 |
|  |  | c.627A>T | p.Q209H | COSM28770 |
|  |  | c.626A>G | p.Q209R | COSM28760 |
|  |  | c.625_627 CAA>TAT | p.Q209Y | COSM28761 |
|  |  | c.1075G>A | p.V359I | COSM13666 |
|  |  |  |  |  |
| **GNA11** | **NM_002067.2** | 626A>T | Q209L | COSM52969 |
|  |  | 667G>A | V223M | COSM51687 |
|  |  | .547C>T | R183C | COSM21651 |
|  |  |  |  |  |
| **NRAS** | **NM_002524.4** | c.35_36GT>AG | p.G12E | COSM144577 |
|  |  | c.35G>A | p.G12D | COSM564 |
|  |  | c.35G>T | p.G12V | COSM566 |
|  |  | c.35G>C | p.G12A | COSM565 |
|  |  | c.34_35GG>AA | p.G12N | COSM12723 |
|  |  | c.34G>A | p.G12S | COSM563 |
|  |  | c.34G>T | p.G12C | COSM562 |
|  |  | c.34G>C | p.G12R | COSM561 |
|  |  | c.37G>C | p.G13R | COSM569 |
|  |  | c.38G>T | p.G13V | COSM574 |
|  |  | c.37G>T | p.G13C | COSM570 |
|  |  | c.38G>C | p.G13A | COSM573 |
|  |  | c.183A>C | p.Q61H | COSM586 |
|  |  | c.182_183AG>GG | p.Q61R | COSM33693 |
|  |  | c.183A>T | p.Q61H | COSM585 |
|  |  | c.181C>G | p.Q61E | COSM581 |
|  |  | c.181C>A | p.Q61K | COSM580 |
|  |  | c.181_182CA>TT | p.Q61L | COSM12725 |
|  |  | c.181_182CA>AG | p.Q61R | COSM579 |
|  |  | c.182_183AA>TG | p.Q61L | COSM30646 |
|  |  | c.182A>G | p.Q61R | COSM584 |
|  |  | c.182A>C | p.Q61P | COSM582 |
|  |  | c.182A>T | p.Q61L | COSM583 |
|  |  | c.52G>A | p.A18T | COSM577 |
|  |  | c.183A>G | p.Q61Q | COSM587 |
|  |  | c.180_181AC>TA | p.Q61K | COSM12730 |
|  |  | c.203G>C | p.R68T | COSM43344 |
|  |  | c.176C>A | p.A59D | COSM253327 |
|  |  | c.149C>T | p.T50I | COSM222543 |
| **c-KIT** | **NM_000222.2** |  |  |  |
|  |  | c.1696A>G | p.N566D | COSM1273 |
|  |  | c.1657T>A | p.Y553N | COSM133763 |
|  |  | c.1676T>A | p.V559D | COSM1252 |
|  |  | c.1676T>G | p.V559G | COSM1253 |
|  |  | c.1676T>C | p.V559A | COSM1255 |
|  |  | c.1679T>A | p.V560D | COSM1257 |
|  |  | c.1706T>G | p.V569G | COSM28037 |
|  |  | c.1669T>C | p.W557R | COSM1219 |
|  |  | c.1727T>C | p.L576P | COSM1290 |
|  |  | c.1965T>G | p.N655K | COSM235682 |
|  |  | c.1924A>G | p.K642E | COSM1304 |
|  |  | c.2458G>T | p.D820Y | COSM12710 |
|  |  | c.2447A>T | p.D816V | COSM1314 |
|  |  | c.2446G>C | p.D816H | COSM1311 |
|  |  | c.2467T>G | p.Y823D | COSM18681 |
|  |  | c.2485G>C | p.A829P | COSM13172 |
|  |  | c.2466T>A | p.N822K | COSM1321 |
|  |  | c.2558G>A | p.W853* | COSM133767 |
|  |  |  |  |  |
| **KRAS** | **NM_033360** | c.35G>T | p.G12V | COSM520 |
|  |  | c.35G>A | p.G12D | COSM521 |
|  |  | c.182A>T | p.Q61L | COSM553 |
|  |  | c.34G>A | p.G12S | COSM517 |
|  |  | c.34G>C | p.G12R | COSM518 |
|  |  |  |  |  |
| **MEK1** | NM_002755.2 | c.370C>T | p.P124S | COSM235614 |
| **MAP2K1** |  | c.332T>G | p.I111S | COSM1238027 |
|  |  | c.607G>A | p.E203K | COSM232755 |
|  |  | c.790C>T | p.P264S | COSM224488 |
|  |  | c.371C>T | p.P124L | COSM1315861 |
|  |  |  |  |  |
